# Assessment of intestinal macromolecular absorption in young piglets to pave the way to oral vaccination: preliminary results

**DOI:** 10.1007/s11259-021-09831-1

**Published:** 2021-09-24

**Authors:** Brodie Deluco, Heather L. Wilson

**Affiliations:** Vaccine and Infectious Disease Organization (VIDO), University of Saskatchewan, 120 Veterinary Road, Saskatoon, SK S7N 5E3 Canada

**Keywords:** Pig, Small intestine, Epithelium, Endosome, Vacuole, Lysosome, Newborn

## Abstract

The small intestine of the piglet has evolved to be permeable immediately after birth to facilitate the uptake of colostrum-derived immunoglobulins as well as other macromolecules, and cells. However, the precise timing of gut closure in today’s precocious pig is not known. We gavaged piglets immediately after birth and at 1-h after birth with Cy5-labeled Ovalbumin (Cy5-Ova) then harvested their small intestine’s 6–7 h later. To assess localization of Cy5-Ova in the small intestinal epithelial cells, we performed immunohistochemistry using a basolateral surface marker and a recycling endosome marker called pIgR, the late endosomal marker Rab7, and the lysosomal marker LAMP-1. Cy5-Ova co-localized with Rab7 and LAMP-1 in the duodenum and jejunum of 0-h old and 1-h old gavaged piglets, but only in the ileum of 0-h gavaged piglets. These data suggest that movement of Cy5-Ova through the late endosomes to the lysosomes was much reduced in the ileum of 1-h gavaged piglets. Cy5-Ova was largely present in epithelial cell digestive and transport vacuoles, but it did not colocalize with pIgR-positive endosomes in 0-h and 1-h gavaged piglets. Differences in macromolecular uptake across the different regions of the small intestine after only 1-h may be due to prior processing of colostral macromolecules, changes in the intestine due to initiation of colonization by microflora and/or the initiation of gut-closure. Understanding the relationship between the localization of Cy5-Ova and small intestinal permeability may contribute to establishing whether oral vaccination in the newborn can capitalize on the transient permeability before gut closure to promote immune protection.

## Introduction

Fetal and newborn piglet intestinal enterocytes possess an apical canalicular system which allows for the production of cytoplasmic vacuoles of various sizes, which are vital for colostrum uptake (Skrzypek et al. 2018). These fetal-derived enterocytes have large vacuoles (referred to as vacuolated fetal enterocytes (VFEs) that absorb and transport macromolecules either to the basolateral surface where they are expelled, or to the lysosomes where they are digested (Skrzypek et al. 2018, 2007). VFEs are first formed in the duodenum in the pig fetus. In the second trimester of pregnancy, VFEs become redistributed towards the jejunal and ileal regions of the small intestine (Smith and Jarvis 1978; Olszewski et al. 2021). VFEs can non-selectively absorb high molecular weight substances by pinocytosis or endocytosis at the apical area of the enterocyte (Fujita et al. 2007; Michael Danielsen and Hansen 2016) but only for a short time after birth (Sangild 2003; Salmon 2012). VFEs are comprised of giant transport vacuoles that disappear 2–3 days after birth and giant digestive vacuoles that are present for up to 3 weeks of age (Baintner 2007, 1994) until VFEs are replaced by adult-type enterocytes that lack an apical canicular system (Skrzypek et al. 2007). Transport vacuoles are formed immediately apical to the nucleus of the enterocyte after newborn piglets consume colostrum and the macromolecules are taken up via endocytosis. The transport vacuoles then migrate to the basolateral area of the cell where the majority of macromolecules, including IgG, completely bypass the Golgi cisternae to release the luminal substances into the intercellular space via exocytosis with preservation of their biological activity (Baintner 2007, 1994; Rodewald and Kraehenbuhl 1984; Burton and Smith 1977; Zabielski 1998). In contrast, digestive vacuoles are relatively large, formed near the apical regions of the cell, and do not migrate (Baintner 2007, 1994). These digestive vacuoles contain nutrients from colostrum and milk that are decomposed into their base components due to enzymes released by connecting lysosomes (Baintner 1994).

Enterocytes have a basolateral and an apical domain which is critical for epithelial cell homeostasis and function. Cellular homeostasis is dependent on the internalization of small solutes, macromolecules, and plasma membrane receptors driven by endocytosis. Endocytosis is mediated by a complex interplay of Rab GTPases that function by regulating epithelial membrane trafficking as well as tethering and budding of vesicles at different locations within epithelial cells (Homma et al. 2019; Gillingham et al. 2014). Rab7 regulates late endosomal membrane fusion and trafficking in the perinuclear region via the interaction of Rab7-RILP-dyenin-dynactin for the biogenesis and maintenance of the lysosomal compartment (Zhang et al. 2009).

Lysosomes are the terminal degradative compartments of cells and they contain hydrolytic enzymes such as acid hydrolases that degrade cell debris into precursor molecules for macromolecule synthesis. Lysosomal-associated membrane protein 1 (LAMP-1) is a highly *N*-glycosylated protein which is transported from the trans-Golgi network to lysosomes via endosomes (Wilke et al. 2012; Xu et al. 2012). It has a short C-terminal tyrosine-based sorting signal which binds the medium subunits of clathrin adaptor protein-1 (AP-1) and AP-2 resulting in intracellular sorting of lysosomal membrane proteins into clathrin-coated vesicles at the cell surface (Honing et al. 1996; Janvier and Bonifacino 2005). AP-1 regulates the basolateral fusion of lysosomes and, together with vesicle-associated membrane protein 7 (VAMP7), synaptosome associated protein 23 (SNAP-23), membrane cholesterol, and syntaxin-4 regulates lysosomal exocytosis (Xu et al. 2012; Samie and Xu 2014).

Polymeric immunoglobulins (pIgs) are synthesized from *lamina propria* plasma cells in the small intestine. They contain a J-chain and a small acidic polypeptide which connects two IgAs to form dimeric IgA, also known as pIgA (Strugnell and Wijburg 2010; Asano and Komiyama 2011). pIgA binds to the transmembrane pIgR on the basolateral surface of the polarized intestinal epithelial cell (IEC) and the pIgR-pIgA complex is internalized into the basolateral early endosome followed by the microtubule-dependent delivery of the pIgR-pIgA complex to the common recycling endosome (CRE) (Verges 2016; Verges et al. 2004). The pIgR-pIgA complex then travels to the apical surface of the cell within a series of tubules and vesicles from specialized subdomains of the CRE where it fuses with the apical plasma membrane and is expelled (Strugnell and Wijburg 2010; Verges 2016; Verges et al. 2004).

We intended to discern the localization of Cyanine-5 labeled ovalbumin (Cy5-Ova) consumed immediately after birth (0-h old gavaged piglets) and 1-h after birth (1-h old gavaged piglets) using a series of markers pertaining to polarized IECs including pIgR (basolateral surface or endosome marker), Rab7 (late endosomal marker), and LAMP-1 (lysosomal marker). Ultimately, we intend to devise an oral vaccine to be administered to piglets as neonates because the intestine is semi-permeable at this time. Because it is not feasible to expect producers to be present at the birth of all piglets to administer an oral vaccine, we need to establish whether the uptake of antigen across the gut wall is different over a reasonable time frame. We opted to compare whether antigen uptake differed in the localization within the small intestine in piglets that are 0-h old and 1-h old. Colonization by microflora and degree of ingestion of colostrum was not controlled. We used markers of the plasma membrane, vacuoles, and organelles to better appreciate where internalized Cy5-OVA goes in the cells as a means to appreciate the mechanism of uptake.

## Materials and methods

### Animal use and ethics

This work was approved by the University of Saskatchewan's Animal Research Ethics Board and adhered to the Canadian Council on Animal Care Guidelines for humane animal use. All sows are Cambrough Plus genetic Line ( Landrace x Large White). Intrauterine growth restricted piglets are characterized using primary parameters such as steep/dolphin-like forehead, narrow hind part, and birth weight (Bahnsen et al. 2021). All piglets in this study exhibited normocephalic head shapes along with non-narrow hind parts which indicates that the piglets assessed were not IUGR. Piglets were not assessed for weight in this study (Table 1).

**Table 1:** Parity and litter information from sows

Sow ID	Parity	Piglets born alive	Still born or mummified
91-1F	3rd	11	6 + 2
193-3E	4th	14	1 + 0
713-8F	1st	17	0 + 4

### Labeling of ova with Cy5

Ovalbumin from chicken egg white (Ova) (Sigma-Aldrich Canada Ltd, Oakville, ON; A5503) was labeled with Cyanine-5 (Cy5) reactive dye (Ambion/ThermoFisher Scientific, Burlington, ON, Canada; 5831G). The following formula was used to determine the amount of Cy5 needed for labeling: 8 × molecular weight (MW) of Cy5 x (amount of Ova)/ MW of Ova. Each Cy5 tube was re-suspended in 100 μl of dimethyl sulfoxide (DMSO; Sigma-Aldrich; D2650). A 1:10 ratio of Cy5 dye to protein and 0.3 M sodium carbonate buffer (Sigma-Aldrich) were incubated overnight at 4 °C with nutation and then placed on top of a 3 K Amicon centrifugal filter (ThermoFisher Scientific) before centrifugation at 16,000 × g for 10 min. After centrifugation, filters were inverted, and samples were washed 4 times with distilled water. The filters were then placed in new microcentrifuge tubes and then centrifuged at 1000 × g for 2 min to dispense the Cy5-labeled Ova.

### Degree of labeling of Cy5 to Ova

To calculate the degree of labeling of Cy5 to Ova, absorbance values of Cy5 at A_280_ and A_555_ were first calculated with a Biochrom Spectrophotometer (Libra S22, MBI Lab Equipment, Kirkland, QC). Next, the protein concentration was calculated using the molar extinction coefficient of Ova. Finally, the moles of Cy5 per moles of Ova were calculated using the molar extinction coefficient of Cy5.

### Tissue collection

Piglets were randomly selected from 3 litters immediately after birth, marked, and fed 300 mg of Cy5-Ova suspended in a total volume of 14.2 ml phosphate-buffered saline (PBS; Sigma-Aldrich) with a gavage tube gently inserted into their stomachs (termed “0-h old gavaged”, n = 3, one per litter). Other piglets were marked immediately after birth; however, they were not fed 300 mg of Cy5-Ova until 1-h after birth (termed “1-h old gavaged”, n = 3, one per litter). A 1-h old control piglet that was not gavaged with Cy5-Ova was also selected. All piglets were left to suckle from their sows for another 5 h. The piglets were then humanely euthanized with a Zephyr pneumatic stunner coupled with exsanguination. The Zephyr pneumatic stunner is a non-penetrating captive bolt device that uses concussive force to render piglets unconscious without pain.

Duodenum, jejunum, and ileum intestinal segments (10–15 cm in length) were obtained and transiently placed within separate 100 ml Erlenmeyer flasks with DMEM media (Sigma-Aldrich) on ice to preserve tissue integrity and to minimize IEC breakdown. Duodenal intestinal segments were excised 10–15 cm distal from the pylorus of the stomach, ileal intestinal segments were excised 10–15 cm proximal from the ileocecal fold. Jejunal intestinal segments were excised at 50% of the length of the small intestine which has been previously used for intestinal analysis in similar studies (Shirkey et al. 2006; Pasternak et al. 2016).

### Immunohistochemistry of intestines

Small cross-Sects. (1 cm^2^) of the intestinal segments from each piglet were placed within tissue-loc biopsy cassettes (ThermoFisher Scientific; 58,931) for immunohistochemistry (IHC) purposes. Tissues were fixed in 10% buffered formalin (Sigma-Aldrich) for 48 h and then dehydrated for 24 h in a series of increasing concentrations of alcohol (EtOH 70%, EtOH 80%, EtOH 95%, EtOH 100%, EtOH 100%—xylene equal mix, and xylene) with the use of a RVG1 tissue processor (Rankin, MI, USA) before embedding in paraffin. Once tissues were embedded in paraffin blocks, a Microm Automatic Microtome (Thermo Scientific) was used to cut 5 μm tissue slices which were carefully placed on Superfrost Plus microscope slides (ThermoFisher Scientific; 22–034-979) before dehydration at 60 °C overnight. Small intestinal tissue sections of 0-h old gavaged and 1-h old gavaged piglets were removed from the oven and de-paraffinized in decreasing concentrations of alcohol (xylene, EtOH 100%, EtOH 95%, and EtOH 70%). Slides were blocked for 3 h at room temperature in 5% (w/v) blotting grade blocker non-fat dry milk (BIO-RAD Laboratories, Hercules, CA, USA; 170–6404) in 1X PBS without magnesium or calcium (PBSA). Next, heat-induced antigen-retrieval (HIAR) was carried out in Tris–EDTA buffer (10 mM Tris, 1 mM EDTA Solution, 0.05% Tween 20, pH 9.0; Sigma-Aldrich) for 13 min at power level 6 within a Panasonic microwave oven (NN-7808).

The following primary antibodies within DAKO antibody diluent (Agilent, Santa Clara, CA, USA; S302283-2) were added to the slides: anti-pIgR antibody (Abcam Inc., Toronto, ON, Canada; ab96196) (1:250 dilution), anti-Rab7 antibody (Abcam Inc., ab50533) (1:200 dilution), and anti-LAMP-1 antibody (GeneTex, Irving, CA, USA; 4E9/11) (1:200 dilution). Slides were then incubated overnight at 4 °C. The following day, slides were washed 3X for 5 min in 1X PBS and then incubated with either 1:500 dilution of Alexa 555-labeled goat anti-rabbit IgG (Southern Biosystems, Birmingham, AL, USA; 4030–02), 1:500 dilution of anti-mouse IgG2b labeled-FITC (Southern Biotech; 1092–02), or 1:500 dilution of anti-mouse IgG1 labeled-FITC (Southern Biotech; 1072–02) in DAKO antibody diluent at 4 °C for 4 h. Information on the primary and secondary antibodies are included in Table 2.

**Table 2:** Primary and secondary antibodies and their targets

Target	Company/Catalogue Number	Dilution of primary antibody	Secondary antibody and fluorescence
Rabbit polyclonal to PIGR	Abcam/ ab96196	1:250	Alexa 555-labeled goat anti-rabbit IgG
Mouse monoclonal to Late Endosome Marker RAB7	Abcam /ab50533	1:200	FITC-labeled anti-mouse IgG2b
Mouse monoclonal to LAMP-1	GeneTex/ 4E9/11	1:200	FITC-labeled mouse IgG1

Slides were washed 3X for 5 min in 1X PBS and then dehydrated for one minute in increasing concentrations of alcohol (EtOH 95%, EtOH 100%, and xylene). Finally, VECTASHIELD Vibrance Antifade Mounting Media with DAPI (H-1800, Vector Laboratories) was added to the coverslips (20 mm X 53 mm; Fisherbrand) and then placed on slides. Intestinal sections were imaged using a Leica SP5 confocal microscope (Leica Microsystems Inc., Concord, ON, Canada). The following imaging was used: DAPI (excitation = 358 nm, emission = 463 nm) with the UV diode laser, 405 nm; FITC (excitation = 495 nm, emission = 517 nm) with the argon laser, 488 nm; Alexa 555 (excitation = 553 nm, emission = 568 nm) with the DPSS laser, 561 nm; and Cy5 (excitation = 646 nm, emission = 664 nm) with the HeNe laser, 633 nm.

**Fig. 1:**
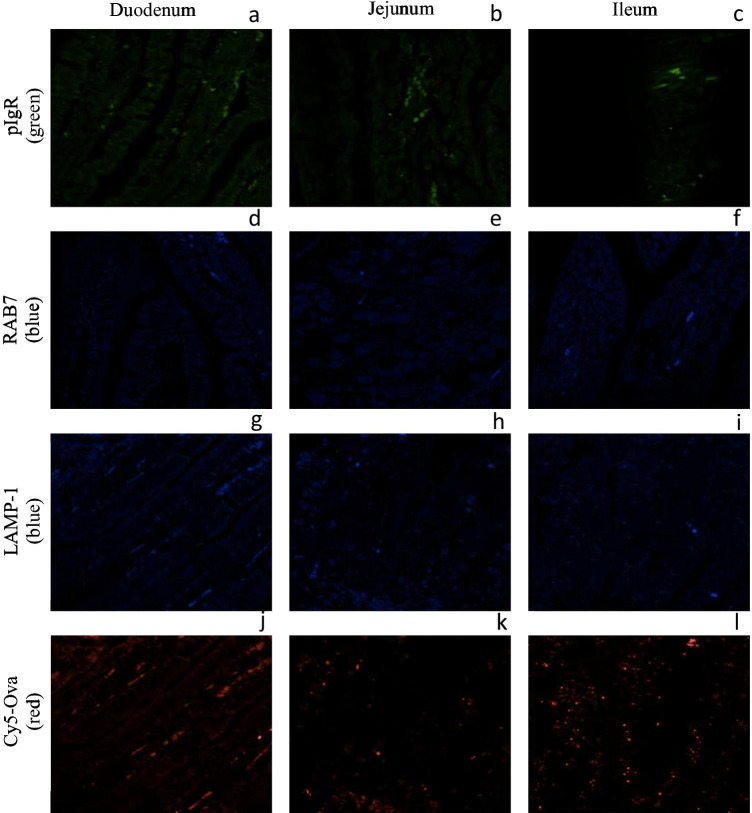
Secondary antibodies for primary antibodies pIgR, RAB7, and LAMP-1, and background fluorescence at 646 nm for Cy5 in the small intestine of a 1-hour old piglet that was not gavaged with Cy5-Ova. These images are representatives of IHC-p performed on duodenum, jejunum, and ileum tissue samples using secondary antibody (A, B, and C) Alexa 555-labeled goat anti-rabbit IgG (H+L) (green), secondary antibody (D, E, and F) anti-mouse IgG2b labeled-FITC (blue), and secondary antibody (G, H and I) anti-mouse IgG1 labeled-FITC (blue). The red colour in J-L are the background fluorescence which corresponds to Cy5 dye (excitation = 646 nm, emission = 664 nm). All images were taken with a Leica Confocal Microscope at 63X

To assess any fluorescence due to non-specific binding of the secondary antibodies and to assess background fluorescence in the fluorescence range for Cy5 dye, intestinal tissues from a 1-h-old control piglet that was not gavaged with Cy5-Ova were incubated with Alexa 555-labeled goat anti-rabbit IgG (green; secondary antibody for anti-pIgR, Fig. 1a-c), anti-mouse IgG2b labeled-FITC (blue; secondary antibody for anti-Rab7, Fig. 1d-f), and anti-mouse IgG1 labeled-FITC (blue; secondary antibody for anti-LAMP-1, Fig. 1g-i). In Figs. 1a-c, there is a very weak, green fluorescent signal in the lamina propria regions indicating very weak background fluorescence or non-specific binding of the Alexa 555-labeled goat-anti-rabbit IgG secondary antibody. In Figs. 1d-f, there is a very weak blue fluorescent signal indicating very weak background fluorescence or non-specific binding of the anti-mouse IgG2b labeled-FITC secondary antibody, again within the lamina propria. In Fig. 1g-i, there is a very weak blue fluorescent signal at the basolateral surface and the lamina propria region indicating very weak background fluorescence or non-specific binding of the anti-mouse IgG1 labeled-FITC secondary antibody.

In Fig. 1j-l, we quantified the fluorescence at 646 nm which corresponds to Cy5 fluorescence, to discern background fluorescence. There appeared to be weak fluorescence in the lamina propria, especially in the duodenal region (Fig. 1j) with fluorescence in the endosomes in the jejunum (Fig. 1 k) and ileum (Fig. 1 l).

### Fluorescence quantification

To quantify the fluorescence of our IHC images, Fiji software was used (a distribution of ImageJ software). For each image, the following measurements were selected: area, minimum and maximum gray value, mean gray value, and integrated density. For each IHC image, three areas of fluorescence and 3 areas with no fluorescence were randomly selected using the freehand selection tool. To calculate the corrected total cell fluorescence (CTCF) for each IHC image, we subtracted the area of the selected region X mean fluorescence of background from the integrated density) (Shihan et al. 2021).

### Statistics

All graphing and statistical analysis was carried out using GraphPad Prism 8 (GraphPad Software, San Diego, CA). Differences between the 0-h old (n = 3) and 1-h old (n = 3) gavaged animals as well as the control animal were analyzed using Kruskal–Wallis One-way analysis of variance (ANOVA) test with Dunn’s multiple comparison’s test. Comparison between the markers and the Cy5-OVA present in the tissues from the 0-h gavaged and 1-h gavaged piglets were compared using Mann–Whitney T-tests. Differences were considered statistically significant if P < 0.05.

## Results

### Immunohistochemistry of intestinal tissues

**Fig. 2:**
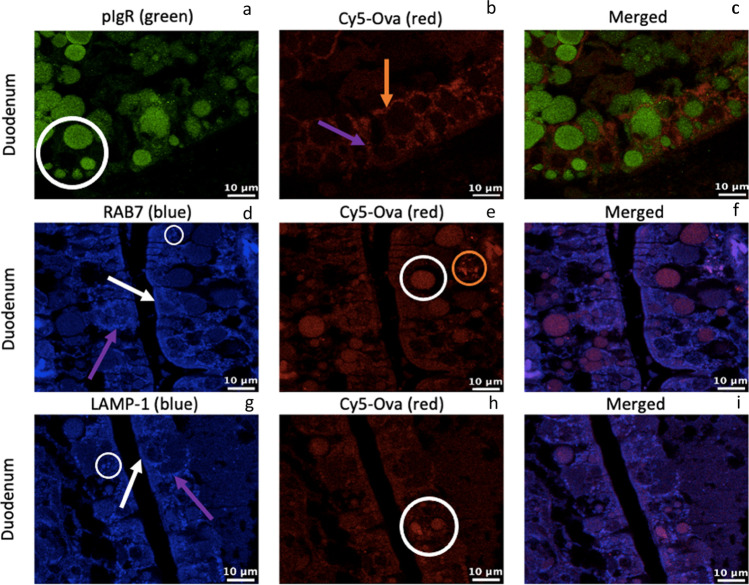
pIgR, RAB7, LAMP-1, and Cy5-Ova localization in the duodenum of a 0-hour old gavaged piglet. These images are representatives of IHC-p performed on duodenum tissue samples. Primary antibodies: rabbit anti-pIgR, mouse anti-RAB7, and mouse anti-LAMP-1. Secondary antibodies: Alexa 555-labeled goat anti-rabbit IgG (green), anti-mouse IgG2b labeled-FITC (blue), and anti-mouse IgG1 labeled-FITC (blue). Cy5-Ova is shown in red. Apical localization is shown with white arrows. Localization between IECs is shown with purple arrows. Basolateral localization is shown with orange arrows. Lamina propria localization is shown with orange circles. Vacuole, endosome, and/or lysosome localization is shown with white circles. All images were taken with a Leica Confocal Microscope at 63X

We performed IHC-p to visualize the presence of pIgR, Rab7, LAMP-1, and Cy5-Ova within the duodenum of 0-h old gavaged piglets (tissue from 1 representative pig is shown throughout). In Fig. 2a, pIgR apepars located within small, medium, and large-sized transport and digestive vacuoles with strong fluorescence throughout the duodenal intestinal epithelium (white circle). Cy5-Ova is located throughout the cell in tiny endsomes most noticably between duodenal IECs (purple arrow) and basolaterally (orange arrow) with moderate fluorescence (Fig. 2b) with no merging of fluorescnece with pIgR (Fig. 2c). Rab7 is present within very small endosomes throughout duodenal IECs (white circle) near the lateral surface between cells (purple arrow) as well as on the apical surface of cells independent of endosomes (white arrow; Fig. 2d). Cy5-Ova is located within large vacuoles in this duodenal section as well as in endosomes throughout the cells (Fig. 2e) where it colocalizes with Rab7 (Fig. 2f). In Fig. 2e, Cy5-Ova also appears to be located within *lamina propria* cells (orange circle) with moderate to strong fluorescence. LAMP-1 is located within lysosomes throughout duodenal IECs (white circle) as well as in lysosomes near the lateral surface between cells (purple arrow). LAMP-1 also appears to be located in lysosomes just beneath the apical surface of the cells (white arrow) (Fig. 2 g,i). In Fig. 2e, Cy5-Ova is located within small, medium, and large-sized transport and digestive vacuoles and in some lysosomes with moderate fluorescence (white circle) where it colocalizes with LAMP-1 (3 h).

**Fig. 3:**
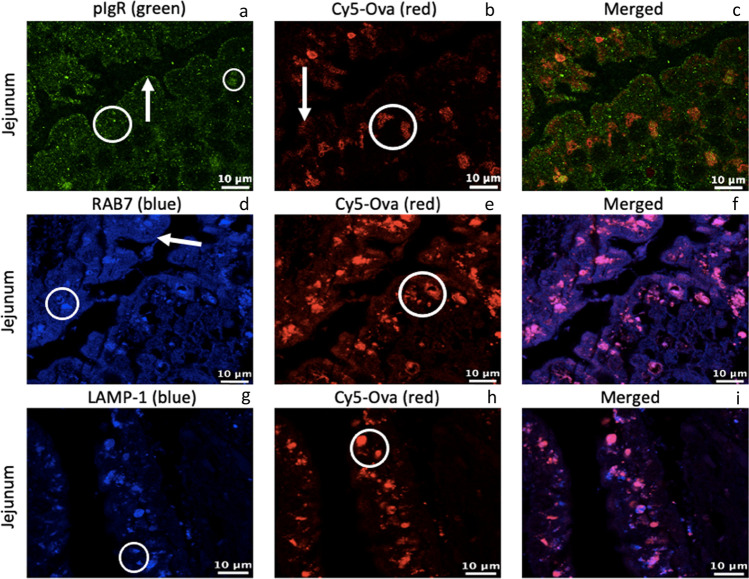
pIgR, RAB7, LAMP-1, and Cy5-Ova localization in the jejunum of a 0-hour old gavaged piglet. These images are representatives of IHC-p performed on jejunum tissue samples. Primary antibodies: rabbit anti-pIgR, mouse anti-RAB7, and mouse anti-LAMP-1. Secondary antibodies: Alexa 555-labeled goat anti-rabbit IgG (green), anti-mouse IgG2b labeled-FITC (blue), and anti-mouse IgG1 labeled-FITC (blue). Cy5-Ova is shown in red. Apical localization is shown with white arrows. Vacuole, endosome, and/or lysosome localization is shown with white circles. All images were taken with a Leica Confocal Microscope at 63X

**Fig. 4:**
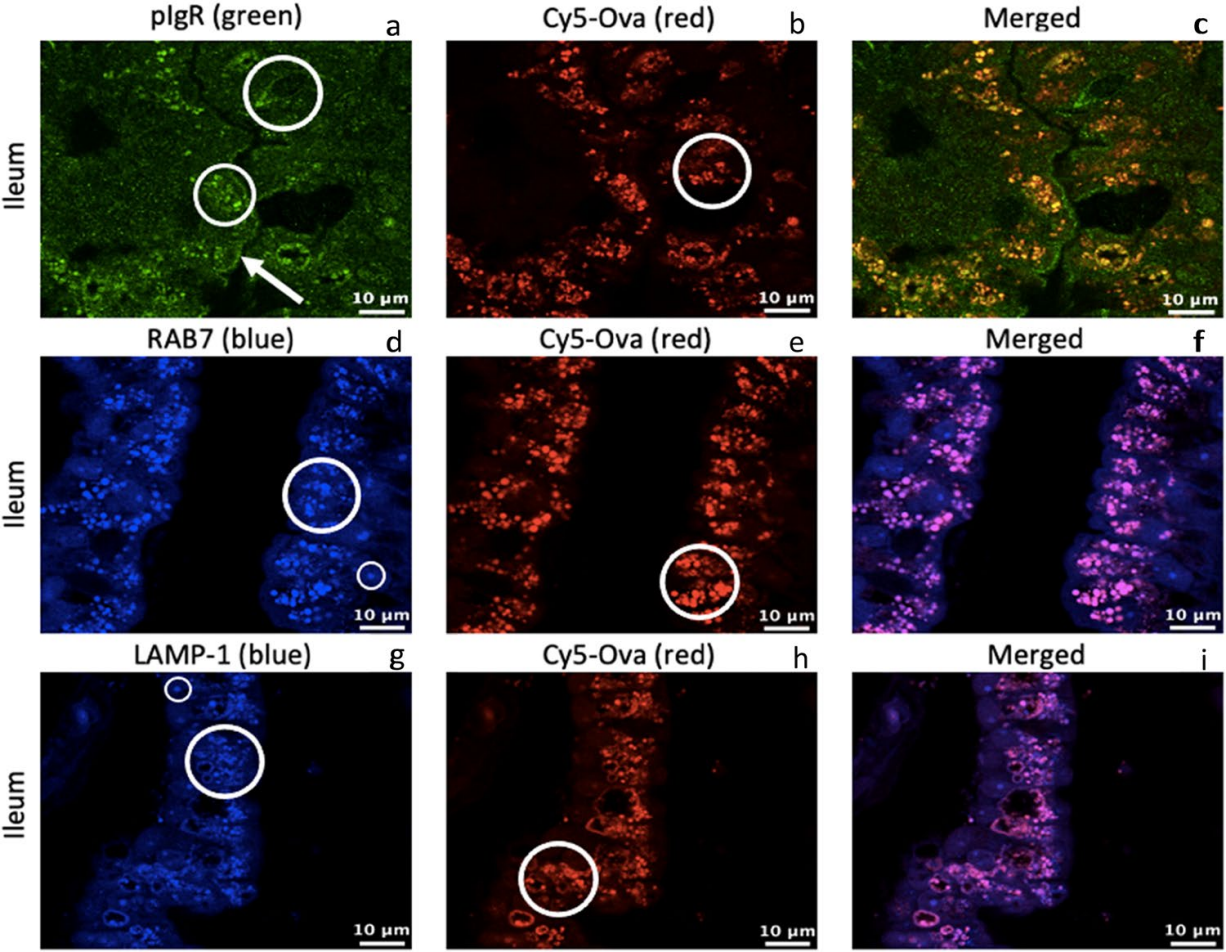
pIgR, RAB7, LAMP-1, and Cy5-Ova localization in the ileum of a 0-hour old gavaged piglet. These images are representatives of IHC-p performed on ileum tissue samples. Primary antibodies: rabbit anti-pIgR, mouse anti-RAB7, and mouse anti-LAMP-1. Secondary antibodies: Alexa 555-labeled goat anti-rabbit IgG (green), anti-mouse IgG2b labeled-FITC (blue), and anti-mouse IgG1 labeled-FITC (blue). Cy5-Ova is shown in red. Apical localization is shown with white arrows. Vacuole, endosome, and/or lysosome localization is shown with white circles. All images were taken with a Leica Confocal Microscope at 63X

We next assessed the presence of pIgR, Rab7, LAMP-1, and Cy5-Ova within the jejunum of 0-h old gavaged piglets (tissue from 1 representative pig is shown throughout). In Fig. 3a, pIgR is located throughout the jejunal IEC endosomes (large white circle), on the apical surface (white arrow) as well as within medium-sized transport and digestive vacuoles near the basolateral surface (small white circle) of the cells. In Fig. 3b, Cy5-Ova is located on the apical surface (white arrow) of cells as well as within medium-sized transport and digestive vacuoles near the basolateral surface of the cells (white circle) independent of pIgR (Fig. 3c). In Fig. 3d, Rab7 is located in endosomes just below the apical surface (white arrow) of jejunal IECs with minimal expression as well as within endosomes throughout the cells (white circle). Cy5-Ova is colocalized with Rab7 within endosomes throughout the jejunal IECs (white circle; Fig. 4e-f). LAMP-1 (Fig. 3 g) and Cy5-Ova (Fig. 3 h) are colocalized within lysosomes throughout jejunal IECs (white circles), however, some lysosomes also appear to be independent of Cy5-Ova (Fig. 3f).

Finally, we show the localization patterns of pIgR, Rab7, LAMP-1, and Cy5-Ova within the ileum of 0-h old gavaged piglets (tissue from 1 representative pig is shown throughout). pIgR (Fig. 4a) and Cy5-Ova (Fig. 4b) colocalize within endosomes throughout ileal IECs (white circles) as well as located on the apical surface (white arrow) of cells and surrounding the apical regions of vacuoles (large white circle). Rab7 (Fig. 4d) and Cy5-Ova (Fig. 4e) colocalize within endosomes thoughout ileal IECs (white circles). Rab7 is also present within endosomes absent of Cy5-Ova (small white circle). LAMP-1 (Fig. 4 g) and Cy5-Ova (Fig. 4 h) colocalize within lysosomes thoughout ileal IECs (white circles) but there also appears to be LAMP-1-positive lysosomes without the presence of Cy5-Ova (small white circle).

**Fig. 5:**
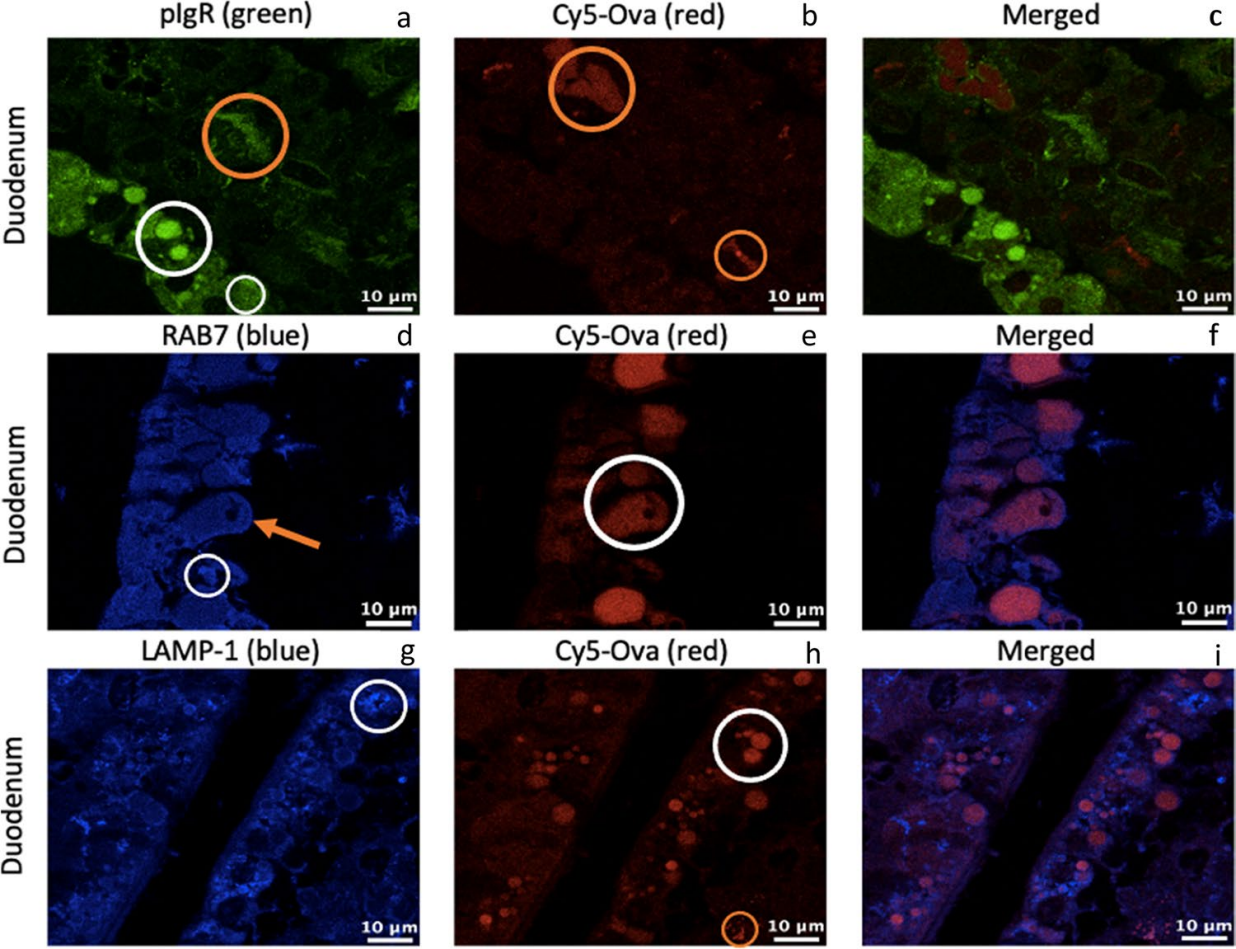
pIgR, RAB7, LAMP-1, and Cy5-Ova localization in the duodenum of a 1-hour old gavaged piglet. These images are representatives of IHC-p performed on duodenum tissue samples. Primary antibodies: rabbit anti-pIgR, mouse anti-RAB7, and mouse anti-LAMP-1. Secondary antibodies: Alexa 555-labeled goat anti-rabbit IgG (green), anti-mouse IgG2b labeled-FITC (blue), and anti-mouse IgG1 labeled-FITC (blue). Cy5-Ova is shown in red. Basolateral localization is shown with orange arrows. Lamina propria localization is shown with orange circles. Vacuole, endosome, and/or lysosome localization is shown with white circles. All images were taken with a Leica Confocal Microscope at 63X

Next, we probed for the presence of pIgR, Rab7, LAMP-1 in the small intestine 6 h post gavage when piglets were gavaged with Cy5-Ova 1-h after birth (tissue from 1 representative pig is shown throughout). In Fig. 5a, pIgR is located within small to medium-sized transport and digestive vacuoles with strong fluorescence throughout the duodenal intestinal epithelium (large white circle) as well as within small endosome (small white circle) and within *lamina propria* cells (orange circles). In Fig. 5b, Cy5-Ova is located within *lamina propria* cells (orange circle) of the duodenal intestinal epithelium with weak to moderate fluorescence. In Fig. 5d, Rab7 is located on the basolateral surface (orange arrow) throughout the cells (white circle) of duodenal IECs. In Fig. 5e, Cy5-Ova is located within large-sized digestive vacuoles with moderate to strong fluorescence and with some evidence of colocalization with Rab7 in endosomes throughout the cell (Fig. 5f). In Fig. 5 g, LAMP-1 is located within lysosomes throughout the duodenal IECs (white circle). In Fig. 5 h, Cy5-Ova appears to be located within small and medium-sized transport and digestive vacuoles with moderate to strong fluorescence as well colocalized within lysosomes (Fig. 5i). Cy5-Ova is also present within duodenal *lamina propria* cells (orange circle; Fig. 5 h).

**Fig. 6:**
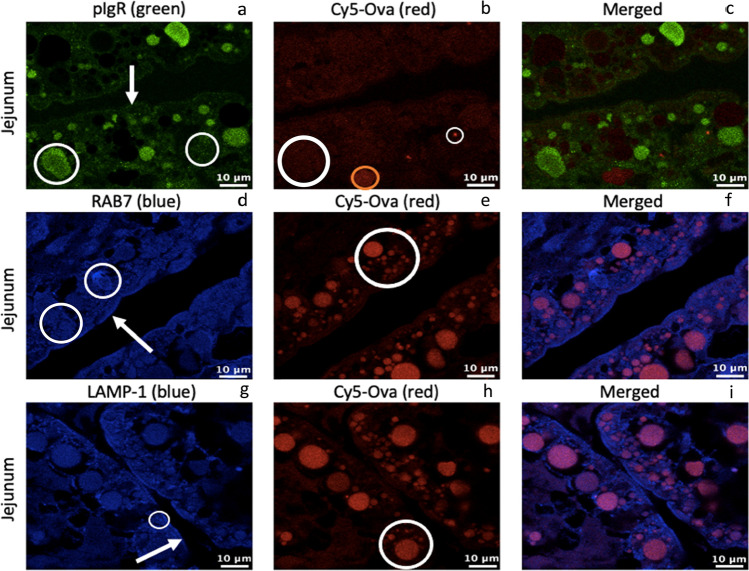
pIgR, RAB7, LAMP-1, and Cy5-Ova localization in the jejunum of a 1-hour old gavaged piglet. These images are representatives of IHC-p performed on jejunum tissue samples. Primary antibodies: rabbit anti-pIgR, mouse anti-RAB7, and mouse anti-LAMP-1. Secondary antibodies: Alexa 555 labeled goat anti-rabbit IgG (green), anti-mouse IgG2b labeled-FITC (blue), and anti-mouse IgG1 labeled-FITC (blue). Cy5-Ova is shown in red. Apical localization is shown with white arrows. Lamina propria localization is shown with orange circles. Vacuole, endosome, and/or lysosome localization is shown with white circles. All images were taken with a Leica Confocal Microscope at 63X

When we investigated the jejunum of 1-h old gavaged piglets (tissue from 1 representative pig is shown throughout), pIgR is located within small, medium, and large-sized transport and digestive vacuoles (Fig. 6a, large white circle) throughout jejunal IECs with medium to strong fluorescence. pIgR also appears to be located on the apical surface (white arrow) and throughout the cells within endosomes (small white circle) with weak fluorscence. In Fig. 6b, Cy5-Ova is located primarily within jejunal *lamina propria* cells (orange circle), however, there appears to be several speckles of Cy5-Ova (small white circle) near the basolateral surface independent of pIgR (large white circle). In Fig. 6d, Rab7 is located on the apical surface independent of endsomes (white arrow) and located throughout jejunal IECs within endosomes (white circle). In Fig. 6e, Cy5-Ova appears to be located within small, medium, and large-sized transport and digestive vacuoles (white circle) throughout the cells largely independent of Rab7. In Fig. 6 g, LAMP-1 appears located on the apical surface independent of lysosomes (white arrow) and located throughout ileal IECs within lysosomes (white circle). In Fig. 6 h, Cy5-Ova appears to be located within small, medium, and large-sized transport and digestive vacuoles (white circle) throughout the cells largely independent of LAMP-1 (Fig. 6i).

**Fig. 7:**
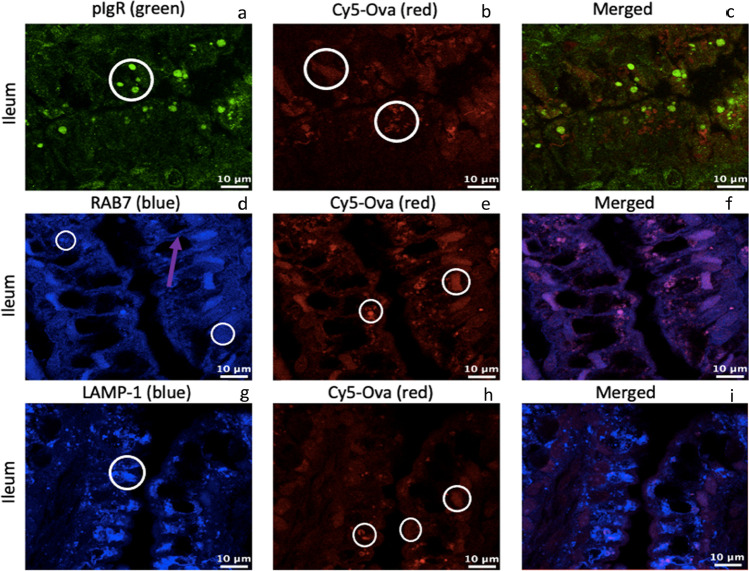
pIgR, RAB7, LAMP-1, and Cy5-Ova localization in the ileum of a 1-hour old gavaged piglet. These images are representatives of IHC-p performed on ileum tissue samples. Primary antibodies: rabbit anti-pIgR, mouse anti-RAB7, and mouse anti-LAMP-1. Secondary antibodies: Alexa 555-labeled goat anti-rabbit IgG(green), anti-mouse IgG2b labeled-FITC (blue), and anti-mouse IgG1 labeled-FITC (blue). Cy5-Ova is shown in red. Central localization between IECs is shown with purple arrows. Vacuole, endosome, and/or lysosome localization is shown with white circles. All images were taken with a Leica Confocal Microscope at 63X

When we investigated the ileum of 1-h old gavaged piglets (tissue from 1 representative pig is shown throughout), we observed that pIgR is located within small to medium-sized vacuoles near the apical regions of ileal IECs (Fig. 7a). In Fig. 7b, Cy5-Ova is located within large-sized transport and digestive vacuoles (white circle closest to the top of the image) that are irregular in shape in comparison to the typical circular nature of vacuoles. Cy5-Ova is also located within endosomes throughout the cells (white circle closest to the bottom of the image) independent of pIgR (Fig. 7c). In Fig. 7d, Rab7 is located within endosomes near the lateral surface between ileal IECs (purple arrow). Rab7 (Fig. 7d,f) and Cy5-Ova (Fig. 7e,f) colocalize within endosomes throughout the cells (small white circle) and within medium to large-sized transport and digestive vacuoles (large white circles) that are an irregular shaped and located near the basolateral region of the cells. Finally, LAMP-1 appears to be located within lysosomes throughout ileal IECs (white circle; Fig. 7 g). Cy5-Ova appears to be located within medium to large-sized transport and digestive vacuoles near the apical and basolateral regions of the cells (the two white circles closest to the right of the image; Fig. 7 h). Cy5-Ova is also located within lysosomes throughout the cells (white circle closest to the left of the image) with LAMP-1 (Fig. 7i).

**Fig. 8:**
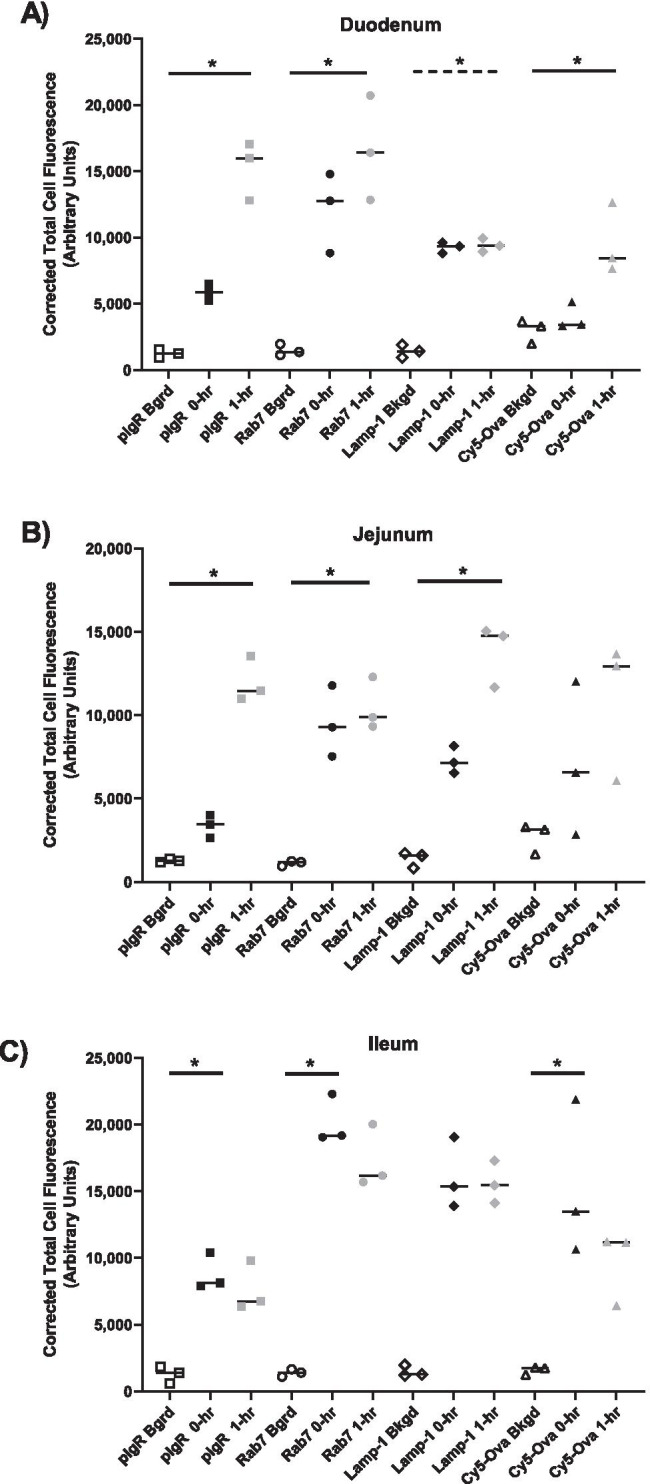
Quantification of fluorescence. Immunohistochemistry was performed on Duodenum (A), Jejunum (B) and Ileum (C) from control, 0 hour gavaged and 1 hour gavaged piglets using rabbit anti-pIgR, mouse anti-RAB7, and mouse anti-LAMP-1 primary antibodies and Alexa 555-labeled goat anti-rabbit IgG(green), anti-mouse IgG2b labeled-FITC (blue), and anti-mouse IgG1 labeled-FITC (blue) secondary antibodies. Cy5-Ova was also quantified. Each symbol represents one animal. Median values are denoted by a horizontal bar and statistical comparisons made to the control animal and the gavaged animals are marked statistical significance using Kruskal-Wallis test with Dunn’s multiple comparisons test. as P<0.05 (*). The dotted line indicates statistical difference with the Kruskal-Wallis test that was not statistically different between groups using Dunn’s multiple comparisons test

When we compare the corrected total cell fluorescence with the 0-h gavaged piglets (Fig. 2,3 and 4) and the 1-h gavaged piglets (Fig. 5, 6 and 7) to the background fluorescence in Fig. 1, we observed that the duodenum and jejunum from the 1-h old gavaged piglets had significantly more pIgR, Rab7, and LAMP-1 (jejunum only) and Cy5-OVA (duodenum only) than the control animal (Fig. 8a,b). In contrast, the ileum of the 0-h old gavaged piglets had significantly more pIgR, Rab7 and Cy-5 relative the control tissue (Fig. 8c). There was not significant difference in the fluorescence between the piglets gavaged at 0-h or 1-h for all tissues, all markers and for Cy5-OVA.

## Discussion

Piglets must ingest colostrum within hours after birth to receive colostrum-derived antibodies and macromolecules for immune system development and protection. It has been previously reported that the small intestine of newborn piglets that are cross-fostered take up colostrum-derived antibodies, macromolecules, and cells (Bandrick et al. 2011, 2014a, 2014b). However, colostral cells from a non-biological sow does not cross the suckling piglet’s intestinal wall (Bandrick et al. 2011; Loving et al. 2014). Furthermore, all segments of the small intestine are reported to lose their abilities to transport macromolecules and cells across the intestinal epithelium 36 h after birth (Sangild 2003; Westrom et al. 1989). This loss of transport is also referred to as “gut closure” and is presumed to occur by decreased endocytotic capabilities of IECs, however, the exact mechanism is currently unknown. In addition, modification of the assembly and composition of tight junction proteins such as claudins which regulate the high-capacity pore pathway between IECs may also contribute to gut closure (Deluco et al. 2021; Pasternak et al. 2015). Tight junctions are a complex of proteins that regulate the passage of small, uncharged solutes and ions between adjacent cells (Madara et al. 1992; Tsukita et al. 2001). Understanding the mechanism by which orally administered antigens traverse the intestinal epithelium immediately after birth may help to provide insight on the mechanism of uptake of an orally administered antigen that is relevant for vaccine development. Visualization of the location of the ingested antigen in relation to endosomal and epithelial cell surface markers may facilitate understanding of the mechanism of antigen transport and processing.

Throughout the small intestine, endocytosis takes place at the microvilli base where there are deep invaginations between the adjacent microvilli (Gonnella and Neutra 1984). Research in the newborn rat ileum showed that enterocytes non-selectively take up macromolecules and process them through a complex array of membrane compartments towards a giant vacuole, which appears to be consistent with a lysosome that is responsible for degradation of milk-derived products (Knutton et al. 1974). Furthermore, macromolecules can be taken up by both receptor-mediated and non-selective endocytosis for degradation in the lysosome or for transport using the transepithelial transport pathway (Knutton et al. 1974; Siminoski et al. 1986). Others showed that radio-labeled proteins introduced to the rat jejunal and ileal epithelium localized to apical endosomal compartments and were also associated with lysosomal vacuoles, suggesting it was targeted for degradation and for transport but that they were also observed at basolateral cell surfaces and lamina propria suggesting transport (Gonnella et al. 1987, 1989). Understanding the mechanisms responsible for route the macromolecules towards transport in the neonatal period has implications for possibly manipulating barrier function.

In the present study, we investigated how Cy5-Ova is taken up and transported within IECs of the 0-h old gavaged and 1-h old gavaged piglet. In duodenal, jejunal, and ileal IECs, Cy5-Ova does not colocalize with pIgR on the surface of the cells or within pIgR + endosomes, regardless of whether they were gavaged with Cy5-Ova pre- or post-suckling. One exception was that co-localization between pIgR and Cy5-Ova was observed within endosomes throughout ileal IECs in 0-h old gavaged piglets. Cy5-Ova appeared to localize within small, medium, and large-sized transport and digestive vacuoles and largely colocalized with Rab7 in the endosomes throughout the small intestine in 0-h gavaged piglets. In contrast, Cy5-Ova colocalized with Rab7 in the ileum but it was largely independent of Rab7 in the duodenum and jejunum in the 1-h gavaged piglets.

Cy5-Ova colocalized with lysosomal marker LAMP-1 in duodenal, jejunal and ileal IECs in 0-h gavaged piglets as well as being present witihin vacuoles. Colocalization with LAMP-1 was not observed within 1-h old gavaged piglets. One possibility is that it takes longer for the piglets that received colostrum to process the gavaged Cy5-Ova and that, given more time, it would be present in the lysosomes in this region of the gut, or that there are region specific differences in localization. Localization of Cy5-Ova within lysosomes of newborn piglets may indicate that the antigen is undergoing degradation and/or that we are simply observing cleavage of the Cy5 dye molecules from the antigen. SDS-PAGE analysis of the processed tissues may elucidate whether Ova is being cleaved from Cy5 in the lysosome or whether it remains covalently associated.

Colocalization observed between Rab7-Cy5-Ova and LAMP-1-Cy5-Ova suggests that Cy5-Ova has entered duodenal IECs via endocytosis and has progressed through early endsomes, recycling endosomes, late endosomes, and localized within lysosomes. To confirm the presence of Cy5-Ova within early endosomes and recycling endosomes, other markers should be explored such as Rab5 (early endosome marker) and Rab25 (recycling marker). Another future direction could be to examine the effects of endocytotic inhibitors on antigen uptake. Chloroquine is an aminoquinolone derivative that is used in the primary treatment of malaria. Chloroquine has also been shown to be an effective inhibitor of clathrin-dependent endocytosis by affecting the function of clathrin and clathrin-coated vesicles (Chen et al. 2009). Filipin is a polyene antibiotic that binds to cholesterol within the epithelial cell membrane thus making it an effective inhibitor of clathrin-independent endocytosis (Dutta and Donaldson 2012). Another technique to assess the localization of ingested antigen may include using BODIPY-conjugated DQ-Ova (a self-quenched conjugate of Ova) (Liu et al. 2017). Proteolytic cleavage of DQ-Ova in the lysosomes of newborn piglets would exhibit brighter fluorescence due to the release of BODIPY dye molecules (Liu et al. 2017). The presence of our antigen within late endosomes and lysosomes suggests that Cy5-Ova is entering polarized IECs via endocytosis. Further analysis should be performed to determine whether an orally administered vaccine is degraded within the lysosomes and whether this negatively impacts antigen presentation and induction of the adaptive immune response.

In pigs, intestinal epithelial cells lack expression of MHCII molecules and therefore cannot act as APCs (Wilson et al. 1996). Therefore, it is critical that vaccines traverse the intestinal wall for oral vaccines to be presented and recognized by the adaptive immune system. Since the intestinal wall is semi-permeable in piglets at birth, this period of time may be used to orally vaccinate pigs, however, it is possible that the neonatal immune system may not be mature enough to respond to the oral vaccine. A previous study examining the effects of orally administered Ova with or without adjuvants in piglets within 6 h of birth (Pasternak et al. 2015b) showed that orally administered Ova induced anti-Ova IgA, IgM, IgG, IgG, and IgG2 antibodies in serum relative to the control piglets gavaged with saline (Pasternak et al. 2015b). These data suggest that the antigen traversed the neonatal gut wall and an adaptive immune response was mounted in the newborn piglet. Further research needs to be performed to establish if an oral vaccine administered when the gut is semi-permeable, prior to gut-closure, can protect against neonatal enteric diseases. Trials should include formulating the vaccine for slow release once it traverses the gut wall to be acted upon by the immune system in the post-neonatal period. Furthermore, there must be confirmation that vaccines administered orally during the neonatal period do not trigger a T regulatory response instead of a stimulatory immune response. While feeding with colostrum has been shown to improve gut function and prevents necrotizing enterocolitis in preterm piglets, it is unlikely that 1-h difference in vaccination in the immediate period after birth would impact gastric emptying, gut maturation or other physiological properties. However, because the precise timing of suckling and the amount of colostrum was not controlled, it is possible that this small difference in gavage timing may have impacted macromolecular absorption (Shen et al. 2015).

Limitations of this research may be the potential rupturing of vacuoles containing Cy5-Ova during IHC processing. During the dehydration process of aqueous formalin-fixed tissues, both transport and digestive vacuoles located within IECs may have ruptured thus releasing their contents in the surrounding cytoplasmic areas. Our research shows the localization of Cy5-Ova within transport and digestive vacuoles located within small intestinal cells exhibit varying degrees of fluorescent intensity. Areas in which vacuoles previously resided appear as black suggesting that these vacuoles may have contained Cy5-Ova but were ‘washed out’ despite the use of formaldehyde, a fixing agent that is generally considered effective in cross-linking proteins and stabilizing the cell matrix. Further, with the exception of the 0-h gavaged pigs, the amount and source of colostrum was not controlled. Gastric emptying rates, microflora composition, etc. were also not controlled. We cannot therefore state that the differences in the uptake of Cy5-OVA between the ages of pigs at time of gavage can be attributed to time alone. A much more extensive analysis with possibly dozens of pigs per group under controlled floral exposure and colostral uptake will need to be performed in the future to clarify this point.

## Conclusions

Our research shows that in duodenal, jejunal and ileal IECs, Cy5-Ova does not colocalize with pIgR on the surface of the cells or within pIgR + endosomes, regardless of whether they were gavaged pre- or post-suckling. Cy5-Ova appears to be largely located within small, medium, and large-sized transport and digestive vacuoles and it was colocalized with RAB7 in the endosomes throughout the small intestine in 0-h gavaged piglets and the ileum of 1-h gavaged piglets. Likewise, Cy5-Ova colocalized with lysosomal marker LAMP-1 in the duodenal and jejunal IECs in 0-h and 1-h gavaged piglets but only in the ileum in the 0-h old gavaged piglets. The intake of colostral macromoleucles takes time to process and therefore appears to reduce the uptake and processing of new antigens in the ileum. Whether the ileal uptake of Cy5-Ova in the 1-h-old gavaged piglets leads to increased transport rather than lysosomal digestion may impact timing of oral vaccine delivery in newborn piglets.

## Data Availability

The datasets used and/or analysed during the current study are available from the corresponding author on reasonable request.
